# Supplementary material for: Changing impacts of Alaska-Aleutian subduction zone tsunamis in California under future sea-level rise
Source: Nat Commun. 2021 Dec 8;12:7119. doi: 10.1038/s41467-021-27445-8 (PMC8655008; doi:10.1038/s41467-021-27445-8)
Supplement: Supplementary file 1 — Supplementary information [file 41467_2021_27445_MOESM1_ESM.pdf]

Supplementary Figures and Tables

Fig. S1. Range of maximum nearshore tsunami heights (MNTH) for select (M_w8.0, M_w8.5, and M_w9.0) earthquakes.
Fig. S2. Tsunami simulation modeling refinement areas.
Fig. S3. Maximum nearshore tsunami heights (MNTH) for uniform, nonuniform, and our method of earthquake slip modeling.
Fig. S4. Convergence of maximum nearshore tsunami heights (MNTH).
Fig. S5. Earthquake recurrence analysis area and b-value calculation.

Table S1. Range of slip and deformation used in earthquake and tsunami simulations.
Table S2. Relative rates of earthquakes along the entire Alaska-Aleutian subduction zone.
Table S3. Distant-source maximum nearshore tsunami heights (MNTH) distributions at the Ports of Los Angeles and Long Beach.

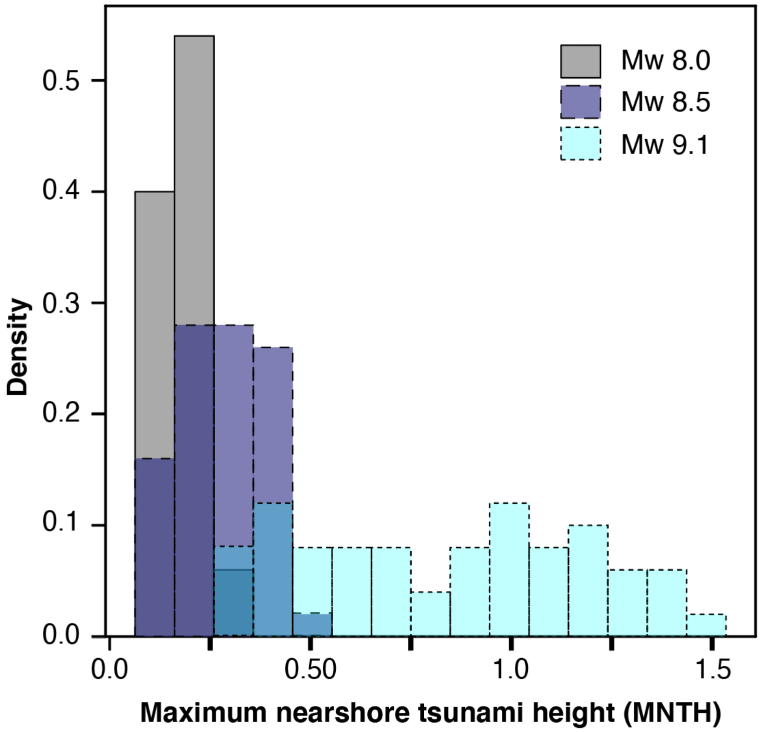

Figure S1. Maximum nearshore tsunami heights (MNTH) at the Ports of Los Angeles and Long Beach generated by tsunamis from M_w8.0, M_w8.5, and M_w9.1 earthquakes.

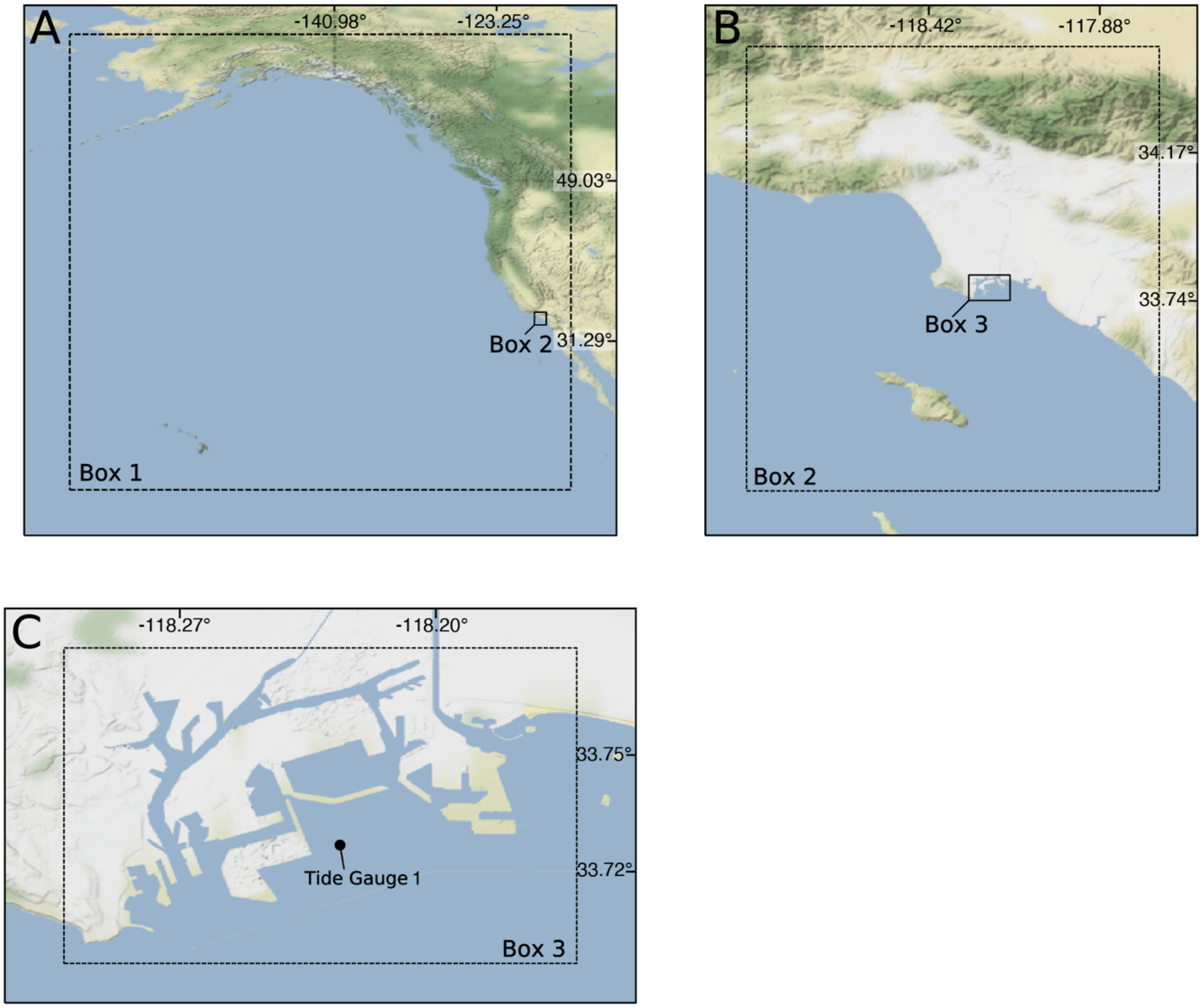

Figure S2. Tsunami simulation modeling refinement areas. Geoclaw resolutions in the A) open Pacific Ocean (1° and 20’); B) deep sea to the continental shelf (20’ and 10”); and C) the Ports of Los Angeles and Long Beach (10” to 1”).

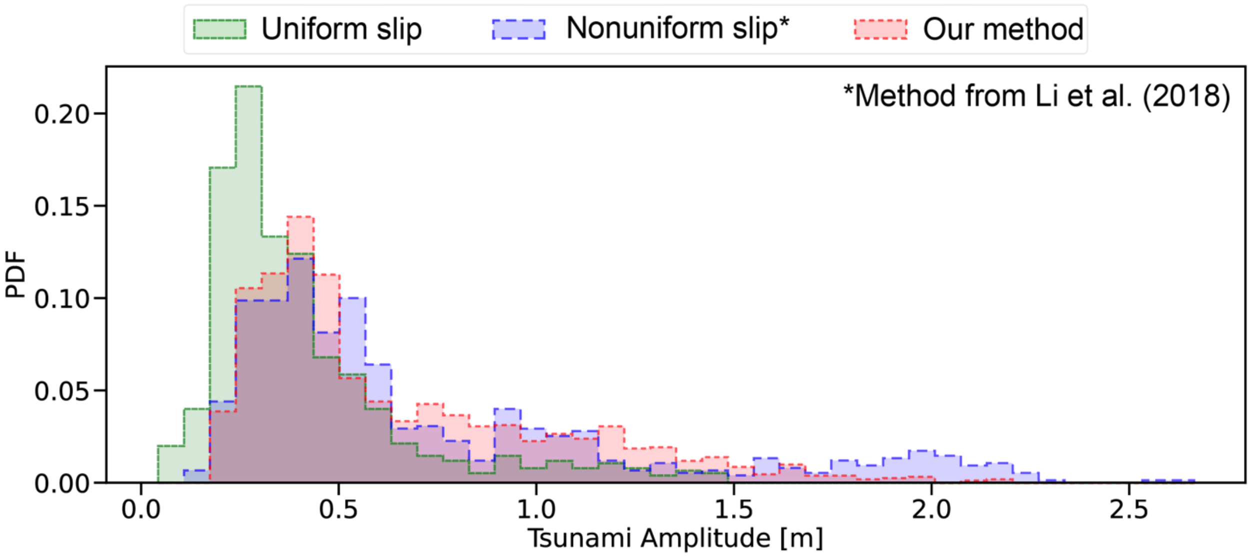

Figure S3. Results of parameter study showing maximum nearshore tsunami height (MNTH) distributions (without tides) for an earthquake modeling approach employing uniform slip (green histogram), nonuniform slip (blue histogram), and our method of varying the number of unit sources over which slip is distributed for each earthquake magnitude (red histogram). Our method produces similar MNTH results to an approach that employs nonuniform slip, although we may underestimate the higher extremes of possible MNTH.

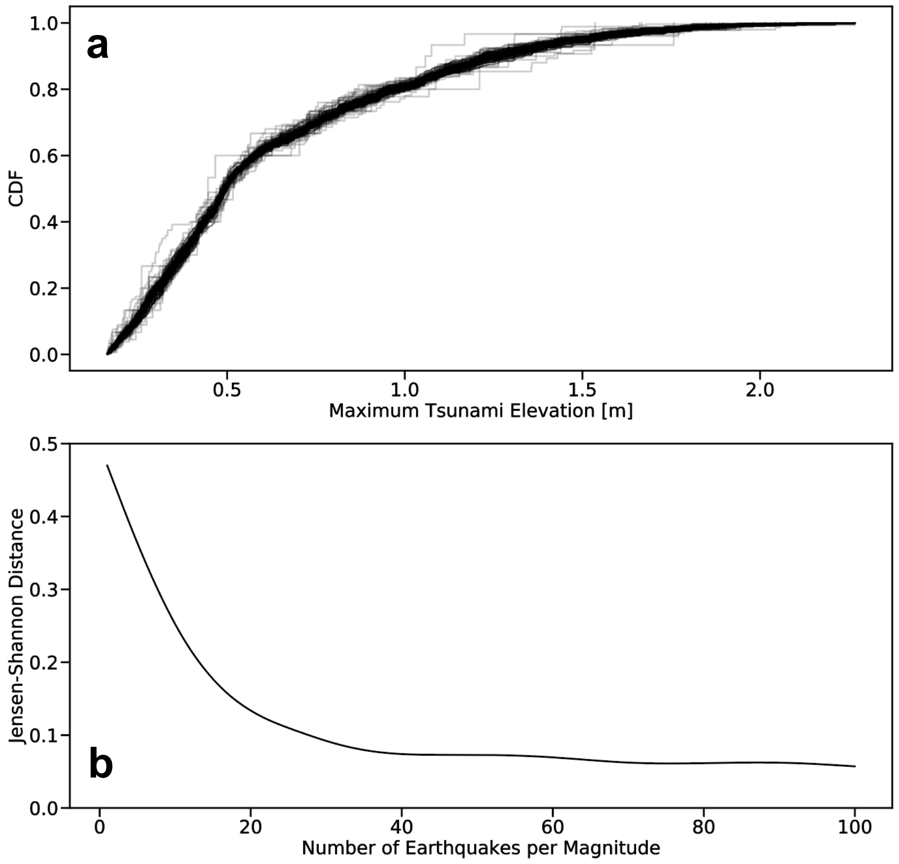

Figure S4. (a) Cumulative density functions of maximum tsunami elevations at the tide gauge for all magnitudes. The different lines (100 total) represent different numbers of earthquakes per magnitude step, from one earthquake per magnitude step to 100 earthquakes per magnitude step. (b) Jensen-Shannon Distance (JSD) as a function of the number of earthquakes per magnitude step showing 50 earthquakes per magnitude step creates a reproducible MNTH at the ports. The Jensen-Shannon distance quantifies how similar (JSD -> 0) or different (JSD -> 1) to distributions A and B are: JSD(A || B). In our case, A(maximum tsunami elevation | N_{EQ} = i - 1) and B(maximum tsunami elevation | N_{EQ} = i) where N_{Eq} is the number of earthquakes per magnitude step. d JSD/d N_{EQ} ~ constant for N_{EQ} > 40.

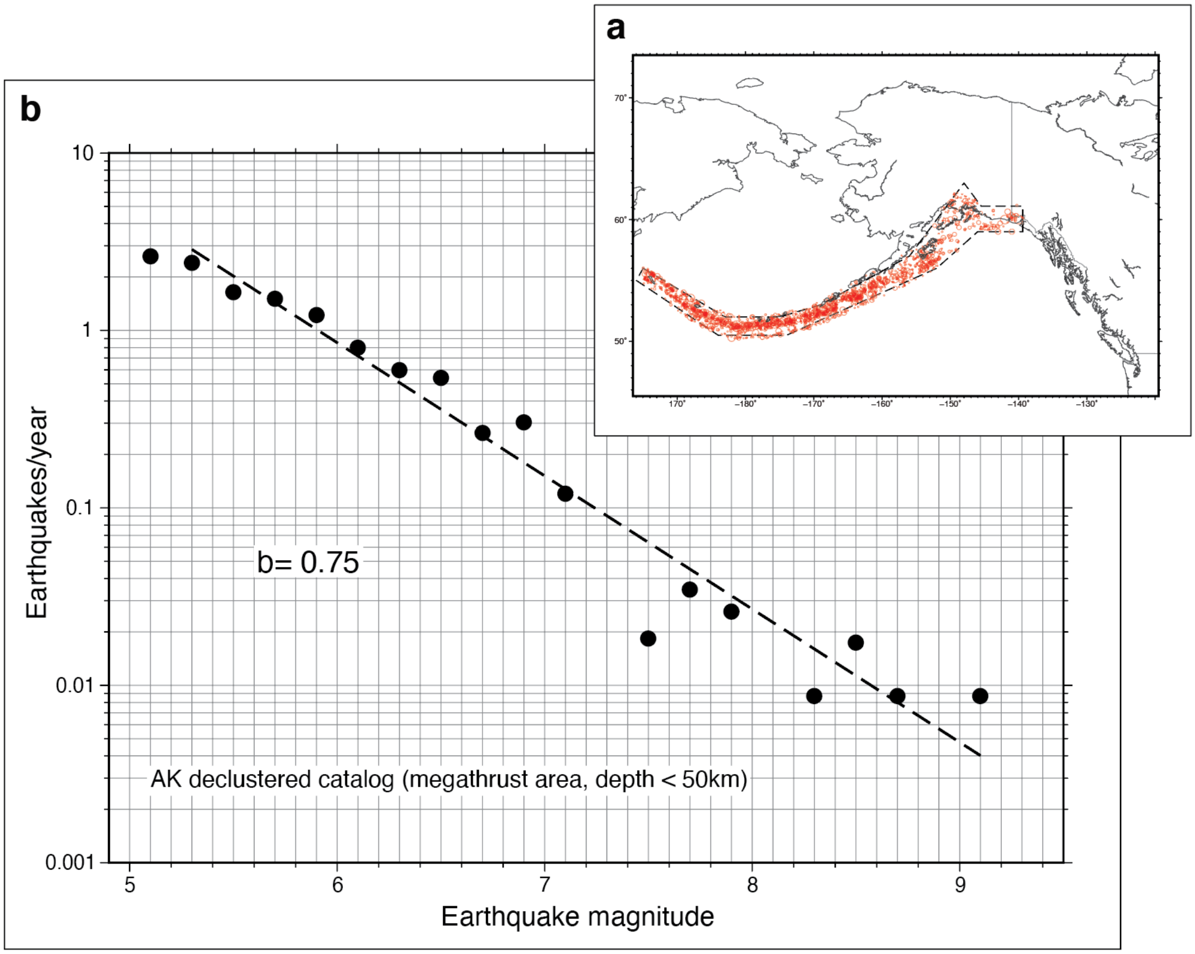

Figure S5. Earthquake recurrence analysis using the Alaska-Aleutian subduction zone USGS declustered catalog a) trimmed to the megathrust area and to depth <50 km. b) Black dots show earthquakes per year in 0.2 magnitude-unit bins, counted from the catalog, assuming completeness levels of M7.4+ since 1900, M7.0+ since 1950, M5.6+ since 1964, and M5.0+ since 1990. The dashed line shows a fit to the counted rates using Weichert's maximum-likelihood method (1980), which gives a b-value of 0.75. The method accounts for the variable completeness; the bin centered at M5.1 is considered incomplete and is not included in the fit.

Tables

Table S1. The range of slip and corresponding deformation used for each earthquake magnitude in our earthquake and tsunami simulations, given in meters.

	**Slip (m)**			**Deformation (m)**		
**M_w**	**Minimum**	**Average**	**Maximum**	**Minimum**	**Average**	**Maximum**
**8.0**	1.87	2.37	2.81	0.84	1.06	1.26
**8.1**	1.59	2.48	3.96	0.75	1.12	1.79
**8.2**	2.24	3.55	5.60	1.00	1.59	2.53
**8.3**	3.16	4.97	7.91	1.41	2.23	3.57
**8.4**	3.94	5.52	7.45	2.56	3.16	4.25
**8.5**	3.94	6.47	10.52	1.85	3.07	5.05
**8.6**	5.57	9.20	14.85	2.47	4.10	6.67
**8.7**	6.99	10.50	15.74	3.10	4.67	7.03
**8.8**	8.08	12.56	22.23	3.77	5.87	9.92
**8.9**	10.47	16.61	25.12	4.89	7.80	11.88
**9.0**	11.83	19.32	29.57	6.68	9.20	13.56
**9.1**	14.74	24.07	41.77	8.33	11.60	19.70
**9.2**	27.23	32.35	50.57	15.40	16.72	22.49
**9.3**	27.78	34.96	55.56	15.54	17.77	25.99
**9.4**	37.17	43.93	70.63	20.52	22.81	32.93

Table S2. Relative rates of earthquakes calculated with a b-value of 0.75 from the declustered catalog of earthquakes along the entire Alaska-Aleutian subduction zone.

Earthquake M_w	Probability of occurrence relative to a <u>Mw8.7</u>	Probability of occurrence relative to a <u>Mw9.0</u>	Probability of occurrence relative to a <u>Mw9.1</u>
**8.0**	3.4	5.6	6.7
**8.1**	2.8	4.7	5.6
**8.2**	2.4	3.9	4.7
**8.3**	2.0	3.4	4.0
**8.4**	1.7	2.8	3.4
**8.5**	1.4	2.4	2.8
**8.6**	1.2	2.0	2.4
**8.7**	1.0	1.7	2.0
**8.8**		1.4	1.7
**8.9**		1.2	1.4
**9.0**		1.0	1.2
**9.1**		0.8	1.0

Table S3. The 95% central range (CR) of MNTH and median of distant-source MNTH distributions including tidal variability at the Ports of Los Angeles and Long Beach for our two emissions pathways (RCP 2.6 and 8.5) and treatments of Antarctic ice sheet uncertainty (K14 and DP16) for 2050, 2070, and 2100. The 2.5th-97.5th percentile range and median of MNTH in the year 2000 are the same for all scenarios (median = 0.62; 2.5th-97.5th percentile range = -0.45-2.03).

		**2050**		**2070**		**2100**	
		**95% CR**	**Median**	**95% CR**	**Median**	**95% CR**	**Median**
**RCP 2.6**	**K14**	-0.27-2.22	0.81	-0.19-2.33	0.89	-0.10-2.52	1.03
	**DP16**	-0.34-2.15	0.74	-0.26-2.26	0.83	-0.17-2.41	0.95
**RCP 8.5**	**K14**	-0.24-2.26	0.84	-0.10-2.44	1.01	0.12-2.81	1.29
	**DP16**	-0.31-2.21	0.78	-0.14-2.60	1.08	0.09-3.92	1.86
